# Outcome of delayed presentation in patients with giant renal cell carcinoma: A case report

**DOI:** 10.1016/j.ijscr.2024.110541

**Published:** 2024-10-29

**Authors:** Nadya Rahmatika, Soetojo Wirjopranoto, Yufi Aulia Azmi, Antonius Galih Pranesdha Putra, Kevin Muliawan Soetanto

**Affiliations:** a Faculty of Medicine, Wijaya Kusuma University, Surabaya, Indonesia; b Department of Urology, Faculty of Medicine Universitas Airlangga – Dr. Soetomo General Academic Hospital, Surabaya, Indonesia; c Department of Health Sciences, University of Groningen, University Medical Center Groningen, Groningen, the Netherlands; d Department of Immunology, Faculty of Medicine Siriraj Hospital, Mahidol University, Bangkok, Thailand; e Department of Biomedical Science, Faculty of Medicine, Universitas Surabaya, Indonesia

**Keywords:** Case report, Renal cancer, Mortality, Survival, Outcome

## Abstract

**Introduction and importance:**

Renal cell carcinoma (RCC) is a common form of malignancy that causes many deaths worldwide. One of the RCC cases that is challenging and requires proper treatment is a large tumor. This report explores the delayed presentation and treatment of giant RCC.

**Case presentation:**

A 53-year-old man came to the referral hospital with a mass in the left pelvis 10 years ago. The patient had been experiencing left flank pain and intermittent hematuria for the past 6 months. Physical examination showed an abdominal mass 20 × 26 cm, cystic consistency, and fixed. A Computed Tomography (CT) scan of the abdomen showed a cystic mass measuring 9.5 × 22.7 × 26.37 cm. The patient underwent a left radical nephrectomy with a chevron incision. The kidney mass was completely removed, and no residual mass or other bleeding was found from the evaluation results. The patient was discharged on the 3rd day after surgery.

**Clinical discussion:**

There was a delay in early detection so the tumor size became large. RCC is considered rare because of its slow growth rate and early detection with imaging techniques. Nephrectomy is the recommended treatment option. All patients should undergo long-term surveillance with routine imaging after nephrectomy to detect recurrent disease early, in this case, the patient is routinely examined every 6 months at follow-up visits.

**Conclusion:**

Once a case of giant renal cancer is diagnosed, immediate action must be taken to avoid patient morbidity and mortality.

## Highlights

• Early management of renal cancer avoids other complications
• Renal cell carcinoma (RCC) is a common form of malignancy that causes many deaths worldwide.
• Adequate management is one of the keys to success in dealing with renal cancer.
• Radical Nephrectomy with chevron incision is the recommended treatment option.

## 1 Introduction

Kidney cancer, which can be defined as renal cell carcinoma (RCC), is a common form of malignancy that causes many deaths each year [1]. RCC accounts for 2.4 % of all cancer diagnoses worldwide. Incidents are projected to increase in the future as more countries turn to Western lifestyles [2]. Kidney cancer globally accounts for more than 131,000 deaths each year and has been found to place a huge economic burden on society [3].

One of the RCC cases that is challenging and requires proper treatment is a large tumor. Giant RCC is an uncommon kidney tumor that grows slowly and has a volume greater than 1000 cc and a diameter greater than 20 cm [4]. The growth rate of giant RCC is 0.06–0.39 cm yearly [5]. There are several problems in diagnosing giant RCC such as relatively low disease prevalence, potential false positives, and slow-growing overdiagnosis [6]. Screening for RCC is recommended if the patient has a heritable syndrome which is known to be associated with the development of RCC [7]. RCC often presents with nonspecific signs and symptoms [8].

Over the past two decades, the death rate from this malignancy has declined due to early detection and treatment [8]. Patients with stage I or II cancer at the time of diagnosis have a five-year survival rate of 80 % to 90 % [7]. Adequate management is one of the keys to success in dealing with renal cancer [9]. There are surgical challenges posed by a tumor of this size. A multidisciplinary approach is required to treat it for good outcomes [10]. Thus, our study explores the delayed presentation and treatment of giant RCC. Reports of this case have been reported in line with the SCARE Guidelines [11].

## 2 Case presentation

**Fig. 1:**
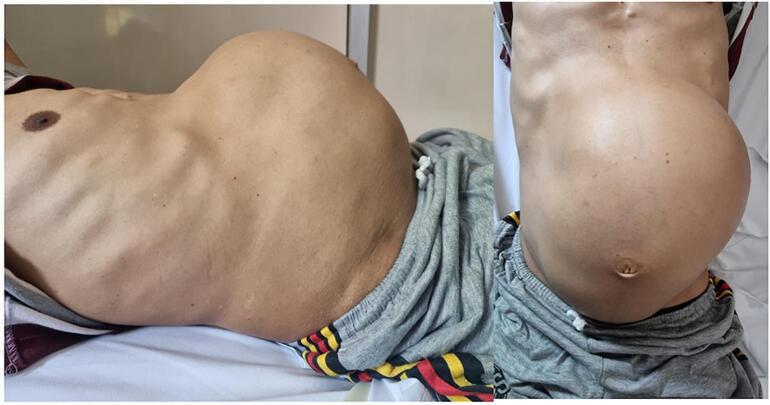
Physical examination.

**Fig. 2:**
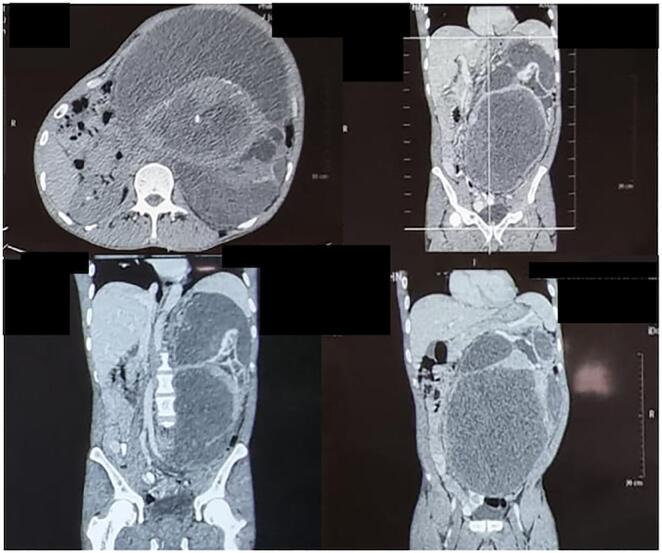
An abdominal computed tomography (CT) scan.

**Fig. 3:**
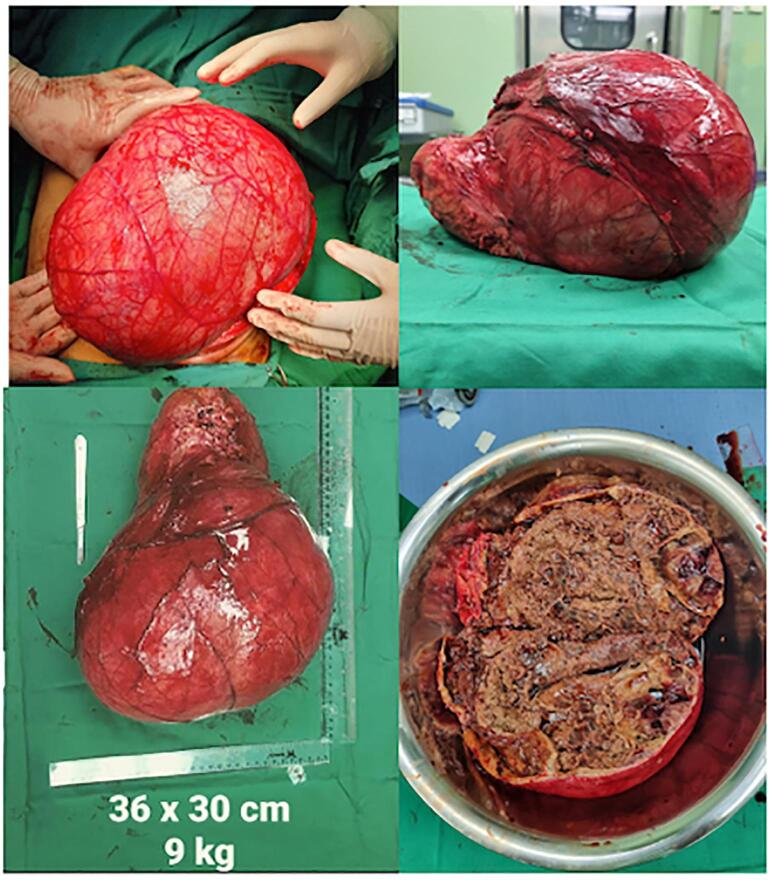
The actual size of the renal mass.

A 53-year-old Male came to the urology polyclinic referred from a first-line hospital with a chief complaint of a mass arising from the left flank 10 years ago. Since 6 months ago, there has been left flank pain and intermittent hematuria. History of other diseases was denied. The examinations carried out are vital signs, physical examination, laboratory examination, and radiology examination. Vital signs showed GCS 456, BP 125/ 85 mmHg, HR: 84 times/ min, RR: 16 times/ min, Temperature: 36.5C, BMI: 19.7 Kg/m2, with Karnofsky score of 90 %. Physical examination showed an abdominal mass of 20 × 26 cm2, cystic consistency, fixed, urine production of 1000 cc/ 24 h. (Fig. 1). Laboratory Examination showed hemoglobin: 12.3 g/dL. Leucocytes: 4.83/ uL, albumin: 3.87 g/dL, serum creatinine: 1 mg/ dL, Calcium: 8.7 mg/dL, LDH 334 u/L. Radiology Examination showed An abdominal computed tomography (CT) scan showed a cystic mass size of 9.5 × 22.7 × 26.37 cm. (Fig. 2). We performed a left radical nephrectomy with a chevron incision. During the operation, 750 cc of blood was lost. The kidney mass was completely removed, and there was no residual mass or other bleeding from the evaluation. The key role in this operation was to free the mass and clamp the vessel. The actual size of the renal mass was 36 × 30 cm with a weight of 9 Kg. (Fig. 3). The post-operative laboratory showed hemoglobin of 9 g/dL. The renal mass was sent to the anatomical pathology department to determine the type. There is no vascular invasion or metastasis in this case. Postoperative care was carried out, including a blood transfusion of packed red cells (PRC), 2 packs/day, until the hemoglobin level reached 10 g/dL. The patient was discharged on 3rd day post-operation. Patient control 1-week post-operation, then routinely control every 6 months.

## 3 Discussion

In this case, there was a delay in early detection so the tumor size had become large and was diagnosed as RCC. The actual size of the renal mass was 36 × 30 cm with a weight of 9 kg after 10 years. Previous studies of kidney tumor growth rates were based on surveillance studies of people with solid kidney masses that had the potential to grow. This is due to the limitation that “significant tumors” often receive prompt treatment and the growth rates of “clinically meaningful” kidney cancers have ultimately been little studied [12]. The growth rate of giant RCC is 0.06–0.39 cm yearly [7]. 45 cases of confirmed RCC with postponed surgical treatment were found in a case study by Zhang et al. (2016) following at least a year of active follow-up. The average tumor size grew during 45.4 months of active surveillance, from 2.39 cm at presentation to 4.54 cm. 36 (80.0 %) tumors had a growth rate of less than 1.00 cm/year, while the mean growth rate was 0.79 cm/year [13]. Patients with untreated renal cell carcinoma had a relatively low progression rate for RCC. Nevertheless, a few cancers developed to the reported stage [14].

In this case, the type of RCC has not been detected. The growth trend of clear cell RCC was faster than that of other histologic subtypes. Certain cases exhibited fast growth kinetics, as demonstrated by positive p53 and high Ki-67 ratio immunohistochemistry indicators. The initial size, gender, or age did not correspond with the RCC growth rate [13]. Other studies have reported no correlation between tumor growth rate and the Fuhrman grading system, gender, histology, or age [15].

There are barriers to the diagnosis of RCC including those related to patient complaints. Vasudev et al. (2019) study reported that in their study, the majority (60 %) of patients were diagnosed incidentally and about one-third had no symptoms when they were diagnosed. The symptomatic presentation was associated with worse outcomes, likely reflecting higher-stage disease [16]. With the increasing use of cross-sectional imaging, kidney masses are increasingly detected when they are still clinically localized. However, most patients still have advanced or locally metastatic disease at the time of diagnosis [17]. The diagnosis of this disease involves imaging techniques such as abdominal ultrasound and CT scan [1]. In this case, an abdominal computed tomography (CT). CT scans have several functions, namely determining the stage of RCC and detecting lymphadenopathy and invasion of the renal vein or inferior vena cava or invasion of adjacent organs [8]. Efforts need to be made to educate patients about common health diagnoses and how to navigate their health to prevent exacerbation or death from the disease [1].

After being diagnosed with a case of giant renal cancer, action must be taken immediately to avoid morbidity and mortality in patients. Management in this case tends to be challenging. Giant RCC surgical resection is difficult [5].Due to RCC's sometimes unusual nature, early detection is difficult, and controlling a major disease that may be too advanced for minimally invasive techniques like laparoscopy or robotic surgery can be a difficult assignment for the surgeon [10]. Active surveillance remains an important component of all renal cell carcinoma management [18]. Several treatment options for RCC can consist of ablation, tumor removal, nephrectomy, and systemic treatment options [1]. In this case, the patient underwent a radical nephrectomy with a chevron incision. Delayed and immediate nephrectomy for renal cell carcinoma provides a comparable long-term overall survival [19]. Other studies have found that certain types of nephrectomy have better outcomes. Laparoscopic radical nephrectomy is associated with better perioperative outcomes compared with open radical nephrectomy. These outcomes include significantly shorter hospital stays, reduced blood loss but not transfusion requirements, reduced postoperative analgesia requirements, and earlier return to normal activities [20].

In this case, the patient has improved. The patient was discharged on 3rd day post-operation. There are no signs of recurrence or residual mass. Other case reports have reported deaths associated with poor preoperative status performance, as well as histopathological sarcomatoid cell results with poor prognosis [4]. In this case, the patient was followed up every 6 months. Based on current European guidelines, all patients undergo a long surveillance program (5–10 years) including regular imaging after a nephrectomy, to detect recurrent disease early on [21].

## 4 Conclusion

After being diagnosed with renal cancer, immediate management must be taken to avoid morbidity and mortality in patients.

## Author contributions

N.R: Conceptualization, Data Curation, Writing-Original draft preparation.

S.W: Conceptualization, Methodology, Data Curation, Investigation, Writing-Original draft preparation, Supervision, Validation.

Y.A.A: Conceptualization, Methodology, Data Curation, Investigation, Writing-Original draft preparation, Supervision, Validation.

A.G.P.P: Writing-Original draft preparation, Writing-Reviewing, and Editing.

K.M.S: Writing-Original draft preparation, Writing-Reviewing, and Editing.

## Consent

Written informed consent was obtained from the patient's family for publication of this case report and accompanying images. A copy of the written consent is available for review by the Editor-in-Chief of this journal on request.

## Ethical approval

Ethical approval for this study was provided by Health Research Ethics Committee of Dr. Soetomo General-Academic Hospital, Surabaya,

## Guarantor

Soetojo Wirjopranoto.

## Funding statement

The author(s) received no financial support for the research.

## Declaration of competing interest

The authors declare no conflict of interest.

## Data Availability

No data was used for the research described in the article.
